# The Effect of Metabolic Syndrome upon the Success of Varicocelectomy

**DOI:** 10.1100/2012/985201

**Published:** 2012-12-23

**Authors:** Ufuk Ozturk, Nevzat Can Sener, Ismail Nalbant, Osman Raif Karabacak, Mustafa Gurhan Ulusoy, M. Abdurrahim Imamoglu

**Affiliations:** ^1^Department of Urology, Ankara Dışkapı Yıldırım Beyazit Education and Research Hospital, Ministry of Health, Ankara 06110, Turkey; ^2^Department of Urology, Yenimahalle State Hospital, Ministry of Health, Ankara, Turkey; ^3^Ankara Training and Research Hospital, Ministry of Health, Ankara, Turkey

## Abstract

We aimed to investigate the impact of metabolic syndrome (MetS) on the varicocele treatment. 101 patients underwent spermatic vein ligation between 2007 and 2010 were retrospectively analyzed. Those patients were divided into two groups as without (*n*: 56, Group 1) or with MetS (*n*: 48, Group 2). All the patients underwent left microsurgical subinguinal spermatic vein ligation. Groups were compared by the improvement on sperm parameters and spontaneous pregnancy rates at a mean of 19 (±4) months followup. When sperm parameters were compared postoperatively, the significant improvement in total sperm count, motile sperm count percentage, and normal sperm percentage was reported. The groups were compared to each other and the improvement seemed significantly better in Group 1. There was no statistically significant improvement difference in the normal sperm percentage between groups. Spontaneous pregnancy rate after two years was 45% in Group 1 and 34% in Group 2 (*P* < 0.05). Patients with MetS and varicocele improved after surgery, but not as well as the similar group without MetS. This may help to show that MetS can be a factor for male infertility.

## 1. Introduction

Metabolic syndrome (MetS) is an important problem in developed countries. In recent years, the prevalence of MetS is growing. The criteria that define MetS can be listed as having hypertension, high low-density lipoprotein (LDL) cholesterolemia, low high-density lipoprotein (HDL) cholesterolemia, hyperglycemia, obesity, physical inactivity, and blood coagulation disorder [1].

MetS can create or aggravate urologic diseases. Its associations with kidney stones [2], benign prostatic hyperplasia [3], and erectile dysfunction [4] are well defined. The association with an important andrologic issue, infertility, is extensively investigated recently [5, 6]. However, it requires further and deeper investigation.

A treatable disease, varicocele, seems to be altered by MetS. We aimed to investigate the impact of MetS on varicocele treatment.

## 2. Patients and Methods

### 2.1. Patients

Patients undergone spermatic vein ligation between 2007 and 2010 were retrospectively analyzed with the local ethics committee approval. Those patients were divided into two groups as without (*n*: 56, Group 1) or with MetS (*n*: 48, Group 2). Color Doppler scrotal ultrasound was performed on all patients. Patients with varicocele were graded according to Dubin and Amelar clinical classification (Grade I: inducible during a Valsalva maneuver; Grade II: palpable; Grade III: visible) system [7]. Color Doppler scrotal ultrasound was performed with 5 to 10 MHz probes during spontaneous breathing and under Valsalva maneuver. The Hirsh testicular Doppler classification (Grade I: no spontaneous venous reflux but inducible reflux with Valsalva maneuver; G II: intermittent spontaneous venous reflux: G III: continuous spontaneous venous reflux) was applied [8]. Group 1 had 30 Grade 2 and 26 Grade 3 varicoceles; Group 2 had 24 Grade 2 and 24 Grade 3 varicoceles. The exclusion criteria were having Grade 1 varicocele, previous infertility treatment (antioxidant drug treatment, hormonal treatment), chronic disease, smoking, hormonal abnormality, and/or previous scrotal surgery.All the patients were performed left microsurgical subinguinal spermatic vein ligation with an operation microscope under 19x magnification (Zeiss, Germany).

The mean patient age was 28.6 (18–31) in Group 1 and 27.8 (19–33) in Group 2. Mean duration of infertility is 13.4 months (12–17) for Group 1 and 14.3 for Group 2 (13–17). Sperm parameters of both groups were summarized in Table 1.

Groups were compared by the improvement on sperm parameters and spontaneous pregnancy rates at a mean of 19 (±4) months followup.

### 2.2. Semen Analysis

In all the patients, semen analysis was performed within 1 h of collection according to the World Health Organization (WHO) guidelines (1999).

### 2.3. Statistical Analysis

Statistical analysis was performed using Statistical Package for Social Sciences (SPSS) 20 software for MAC (SPSS Inc., Chicago, IL, United States). All the data are presented as mean (range). The distribution of the data was evaluated by the Kolmogorov-Smirnov test. The comparisons of the groups were performed using the Mann-Whitney *U* test. *P* < 0.05 was considered statistically significant.

## 3. Results

Patients were evaluated by spermiogram and color Doppler USG after three months. No recurrences were reported. Postoperative spermiogram parameters are summarized in Table 1. When sperm parameters were compared postoperatively, the significant improvement in total sperm count, motile sperm count percentage, and normal sperm percentage was reported. The groups were compared to each other and the improvement seemed significantly better in Group 1. There was no statistically significant improvement difference in normal sperm percentage between groups. Spontaneous pregnancy rate after two years was 45% in Group 1 and 34% in Group 2 (*P* < 0.05).

## 4. Discussion

Metabolic syndrome is a common health problem described with hypertension, increased waist circumference, insulin resistance, and dyslipidemia [1]. It is associated with many urologic diseases, especially andrologic ones. West et al. conducted a study in 2008 and showed a two-fold increase in urolithiasis prevalence in patients with metabolic syndrome [9]. Ekeruo et al. conducted a study and claimed that patients with MetS have stone-forming characteristics such as lower urine pH, gout diathesis, hypocitraturia, and hyperoxaluria [10]. Parallel to these reports, there are several publications about the possible link between MetS and BPH [11–13].

Male infertility and MetS have gained a lot of attention lately. In a multivariate analysis conducted by Bener et al., patients with Type 2 diabetes mellitus and have a BMI > 30 were found to have a three-fold increased risk of infertility [14]. Also, there are other reports claiming a link between BMI and low sperm motility and ejaculate volume [15, 16]. Many hypotheses were formed on this matter. Kasturi et al. showed a proinflammatory state caused by dyslipidemia and suggested impairment in sperm parameters. We believe that our patients had a similar condition to have lower sperm parameters in MetS group. That results are parallel to the current literature.

Varicocele makes a venous accumulation in scrotum and increases oxidative stress and deteriorates spermatogenesis by increasing temperature [6]. There are papers claiming that the same mechanism is possible in patients with MetS. Shafik and Olfat performed scrotal dissection to patients with idiopathic infertility and found scrotal lipomatosis in patients with obesity [17]. This finding may be helpful to explain why our patients with MetS did not improve after surgery as expected.

Another situation for patients with high BMI is the unsuccessfully assisted reproduction techniques (ART). Gopalkrishnan et al. and Bungum et al., revealed a higher risk of infertility for patients with a BMI > 30 [18, 19]. Our data may support the findings since obesity is a part of MetS.

Hyperglycemia, also a criterion for MetS, was proven to have a role in the production of free radicals, which create DNA damage. DNA fragmentation index, which shows DNA damage, was studied several times for the association with MetS [15, 20]. DNA fragmentation index can be determined by Anilin or Toulidin Blue dying. Our study did not cover DNA fragmentation. This is one of the limitations in our study.

In this study, we aimed to show that MetS might be an independent predictor for sperm parameters. Patients with MetS and varicocele improved after surgery, but not as well as the similar group without MetS. This may help to show that MetS can be a factor for male infertility. To our knowledge, our study is one of the first to show the effect of MetS in patients with varicocele. The retrospective nature of the study and low number of patients are the limitations of our study. Prospective studies with higher cohorts may help to support the data obtained.

## 5. Conclusion

MetS is a health problem for developed countries. Its effects on diseases are a new research topic in modern urology. Infertility is one of the most important diseases MetS might cause. We aimed to show its prognostic effect in patients with varicocele. Our study shows a good correlation between MetS and infertility; however, prospective and higher cohort studies should be conducted to support our findings.

## Figures and Tables

**Table 1:** Sperm parameters pre- and postoperatively.

Patient characteristic Mean (±SD)	Group 1	P*	Group 2	P**	P***
Preoperative sperm count (10^6^/mL)	18.01 ± 1.88		17.03 ± 7.6		0.356
Postoperative sperm count (10^6^/mL)	38.40 ± 1.32	0.03	30.1 ± 4.7	0.04	0.04
Preoperative percentage of motile spermatozoa	17.9 ± 8.9		16.3 ± 6.6		0.565
Postoperative percentage of motile spermatozoa	24.7 ± 89.1	0.001	20.6 ± 5.5	0.03	0.03
Preoperative percentage of normal forms	7.01 ± 3.02		5.04 ± 7.05		0.285
Postoperative percentage of normal forms	11.4 ± 2.1	0.001	10.5 ± 4.21	0.001	0.485

P*: comparisons of the preoperative and postoperative values for Group 1.

P**: comparisons of the preoperative and postoperative values for Group 2.

P***: comparisons of postoperative values between groups.
